# Primary Pulmonary Hodgkin's Lymphoma: A Rare Etiology of a Cavitary Lung Mass

**DOI:** 10.7759/cureus.1620

**Published:** 2017-08-28

**Authors:** Halim El Hage, Sami Hossri, Bachar Samra, Dany El-Sayegh

**Affiliations:** 1 Pulmonary and Critical Care Medicine, Staten Island University Hospital; 2 Internal Medicine, Staten Island University Hospital; 3 Department of Hematology and Oncology, SUNY Downstate Medical Center, Brooklyn, NY, USA; 4 Pulmonary and Critical Care Medicine, Staten Island University Hospital, Northwell Health

**Keywords:** primary pulmonary lymphoma, primary pulmonary hodgkin's lymphoma, lymphoma, hodgkins lymphoma, lung mass, pulmonary, primary lung lymphoma, hodgkins

## Abstract

Primary pulmonary Hodgkin's lymphoma (PPHL) is an uncommon disease. This entity is different from Hodgkin’s lymphoma with parenchymal or nodal lung involvement. In this report, we highlight the case of a young female presenting with a six-month history of a productive cough and constitutional B symptoms. Imaging showed cavitary lesions in the right-upper and right-middle lobes. The initial comprehensive infectious workup was negative. Histopathology and immunochemistry confirmed the diagnosis of PPHL. PPHL is an uncommon etiology of cavitary lung lesions. Despite its diagnostic difficulties, awareness of such a disease is crucial, given its high rate of response to treatment, especially in the young population.

## Introduction

Although pulmonary involvement in Hodgkin’s lymphoma is common (15%-40% of cases), primary pulmonary Hodgkin’s lymphoma (PPHL) is a very rarely documented presentation of Hodgkin’s disease consisting of <1% of all lymphomas and even less among primary pulmonary malignancies [1]. Its presentation can sometimes mimic that of certain infectious and inflammatory etiologies, making the diagnostic process challenging. Although cavitations are not uncommon during the course of chemotherapy for PPHL, they are exceptionally rare at the time of diagnosis. We herein report a case of PPHL that presents as a cavitary mass associated with constitutional B symptoms in a young, previously healthy, female.

## Case presentation

**Figure 1:**
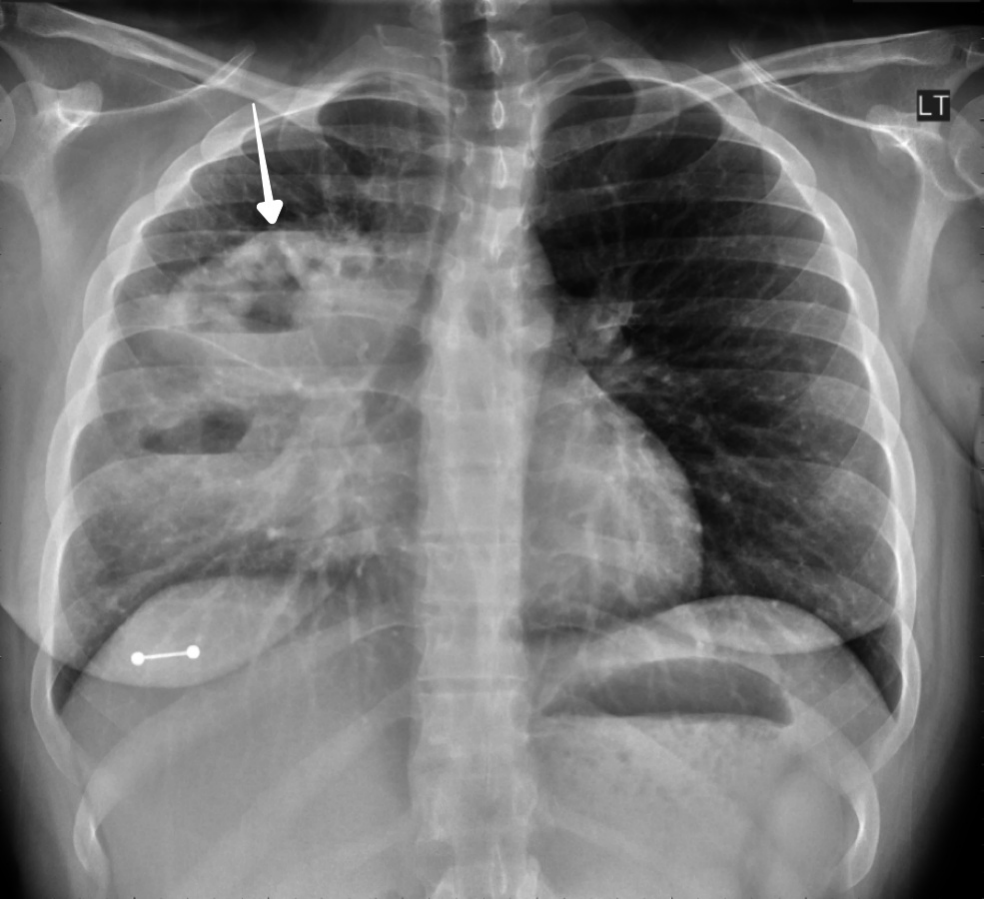
Chest X-ray Chest X-ray (CXR) showing multiloculated cavitary lesions in the right upper and middle lobes, indicated by the white arrow.

**Figure 2:**
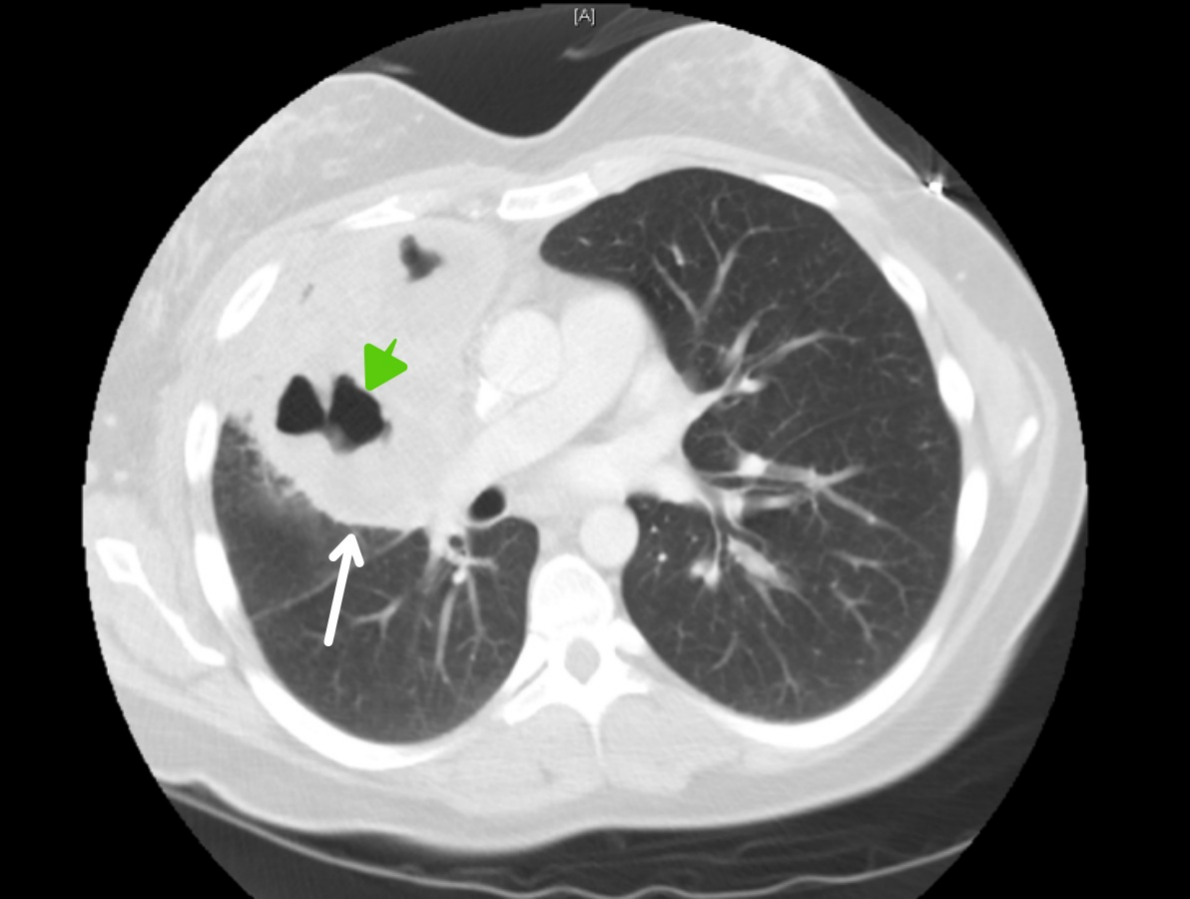
CT chest - axial view This computed tomography (CT) scan of the chest is showing a large heterogeneous mass (white arrow) with internal cavitation (green arrow) in the right middle and upper lobes.

**Figure 3:**
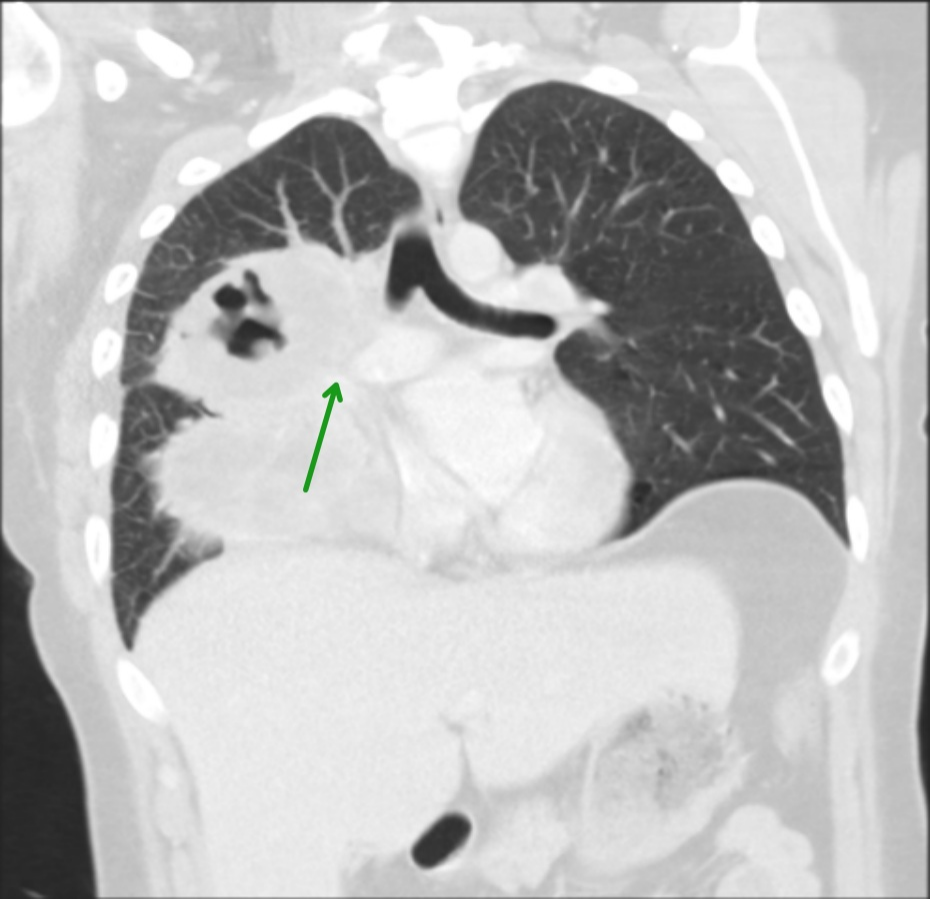
CT chest - coronal view Encasement of the right pulmonary artery (green arrow) is visible.

A 27-year-old female, an active smoker, with no significant past medical history, presented to the emergency department with a worsening productive cough, night sweats, and a 20-pound weight loss over six months. The patient denied recent travel, sick contacts, or illicit drug use and had no personal or family history of malignancies. On presentation, a temperature of 100.1F and a heart rate of 100 beats per minute were recorded. The physical exam was only remarkable for decreased breath sounds in the right upper lung field. She had no palpable lymph nodes or organomegaly. Laboratory workup showed leukocytosis (15,600 mm/L) with a granulocytic predominance. Her comprehensive metabolic panel and lactate dehydrogenase levels were normal. A chest X-ray (CXR) showed multiloculated cavitary lesions in the right upper and middle lobes (Figure 1). This was followed by a chest computed tomography (CT) scan showing a 13.1 x 10.9 x 10.7 cm heterogeneous mass with internal cavitation within the right middle and upper lobes. There were prominent prevascular/mediastinal lymph nodes, measuring up to 1.9 x 1.2 cm. The mass was noted to be encasing the pulmonary artery (Figure 2, 3). The patient was started on ceftriaxone and azithromycin for a presumed community-acquired pneumonia. However, her symptoms persisted and the infectious workup, which included bacterial cultures, acid fast and Gram staining, serum quantiferon, cryptococcal antigen, and human Immunodeficiency virus (HIV) serology, was negative. Bronchoscopy with ultrasound-guided biopsy and bronchoalveolar lavage were nondiagnostic. Therefore, a CT-guided biopsy of the cavitary lesion was performed. Pathology revealed extensive necrosis with rare atypical cells suspicious for malignancy. Immunohistochemistry (IHC) was positive for cluster of differentiation (CD) 30, melanoma ubiquitous mutated protein (MUM), organic anion transporter (OCT) 2, negative for CD20, and indeterminate for B-cell Oct-binding protein (BOB) 1. Paired Box (PAX) 5 was dim. CD15 and CD45 could not be determined since they were obscured by numerous strongly staining background cells, which consisted of a mixture of both B-cells (CD20^+^, PAX5^+^) and T-cells (CD 3^+^). Overall, the morphology and IHC raised the suspicion of classical Hodgkin’s lymphoma (CHL). Given the uncertainty of the pathological diagnosis, our patient underwent a repeat CT-guided biopsy, which confirmed the suspected diagnosis (with the subtype being nodular sclerosing) after additional stains were more supportive of CHL with CD15^-^ and CD45^-^. Staging CT scans of the abdomen and pelvis showed no evidence of extrathoracic disease. Based on the aforementioned findings, a diagnosis of PPHL was made, and the patient was started on doxorubicin, bleomycin, vinblastine, and dacarbazine (ABVD) -based chemotherapy.

## Discussion

PPHL is a rare entity. Only about 100 cases have been reported in the literature since 1927 [2-3]. Typically, three criteria are required in order to make the diagnosis: the characteristic histological features of Hodgkin’s lymphoma, restriction of the disease to the lungs with or without minimal lymph node involvement, and the absence of extrapulmonary disease [4]. Our patient fulfilled all three criteria. Patients can either be asymptomatic or manifest nonspecific systemic and respiratory symptoms [1].

Radiologically, PPHL may mimic pneumonia, lung carcinoma, or metastasis by presenting as a solitary nodule, multiple nodules, or rarely cavitating masses [5]. The latter usually develop throughout the course of chemotherapy, mainly secondary to necrosis. The initial presentation of this case as a cavitary pulmonary lesion is an uncommon manifestation of PPHL.

In contrast to secondary involvement from Hodgkin’s lymphoma, which has a random distribution among the lung parenchyma, PPHL seems to have an anatomical predilection that classically affects the superior portions of the lungs [6]. Given the wide array of radiological and clinical presentations, PPHL can mimic lung carcinomas, granulomatous diseases, as well as infectious processes, most notably tuberculosis. Diagnosis is often delayed due to the nonspecificity of radiological examinations and bronchoscopy findings. In the largest series by Radin et al., only one bronchoscopy out of 39 performed was diagnostic [1]. Diagnosis is established via transthoracic needle aspiration and more invasive methods, such as video-assisted thoracoscopic surgery or open lung biopsy. In fact, some reported cases were confirmed only postoperatively after lobectomy for a suspicious mass. This highlights the difficulty in confirming the diagnosis with a CT-guided biopsy. In our case, our patient had to undergo an endobronchial ultrasound-guided fine needle aspiration and two CT-guided biopsies to finally confirm the diagnosis of classical Hodgkin's lymphoma based on pathology.

The most common histological subtype encountered in PPHL is the nodular sclerosing subtype of Hodgkin’s disease, which is also seen more frequently in women [7]. Survival data remains lacking mainly due to a scarcity of the disease. Nonetheless, a number of prognostic factors have been suggested by experts including the extent of lung involvement, advanced age, the presence of B-symptoms, the number of clinical relapses, and pleural involvement on imaging [3].

Management is variable across the literature, mostly due to a lack of therapeutic guidelines. All three modalities (surgery, chemotherapy, and radiation) have been used depending on the extent of the disease. Surgery has been mostly performed in the setting of an uncertain diagnosis. In those cases, it has been suggested that adjuvant chemotherapy and/or postoperative radiation therapy is associated with better disease control than surgery alone [8].

Generally, it has been accepted that for solitary lesions, surgery and radiation are effective. However, multiagent chemotherapy, with or without radiation, is required for bilateral, diffuse, and more advanced cases with poor prognostic features [3,9]. No consensus exists regarding the best chemotherapy regimen. However, as in Hodgkin's lymphoma, the adriamycin, bleomycin, vinblastine, dacarbazine (ABVD) regimen is preferred over mechlorethamine, vincristine, procarbazine, and prednisone (MOPP) in terms of efficacy and toxicity [10]. The superiority of bleomycin, etoposide, adriamycin, cyclophosphamide, vincristine, procarbazine, and prednisone (BEACOPP) is yet to be determined.

## Conclusions

PPHL is a rare entity that can mimic other pulmonary diseases. The diagnosis is challenging and often delayed due to nonspecific clinical and radiological findings. However, PPHL should be considered as part of the differential diagnosis in patients presenting with lung masses and cavitary lesions. A prompt diagnosis with a lung biopsy is essential in any suspicious case. Awareness of such a disease is of paramount importance due to the high chances of cure with systemic therapy, especially in young adults.
